# In Vivo/In Vitro Properties of Novel Antioxidant Peptide from *Pinctada fucata*

**DOI:** 10.4014/jmb.2006.06002

**Published:** 2020-07-30

**Authors:** Yongkai Ma, Kehui Huang, Yanyan Wu

**Affiliations:** 1 South China Sea Fisheries Research Institute, Chinese Academy of Fishery Sciences; Key Lab of Aquatic Product Processing, Ministry of Agriculture and Rural Affairs of the People’s Republic of China, Guangzhou 50300, P. R. China; 2 Co-Innovation Center of Jiangsu Marine Bio-industry Technology, Huaihai Institute of Technology, Lianyungang, P.R. China; 3 School of Food Science and Engineering, South China University of Technology, Guangzhou 510640, P.R. China; 4 Guangzhou Maritime University, Guangzhou 510725, P.R. China

**Keywords:** *P. fucata*, antioxidant peptide, cellular antioxidant activity, anti-proliferation, molecular docking

## Abstract

Due to the potential of antioxidants to scavenge free radicals in human body, it is important to be able to prepare antioxidant peptides that meet the industrial requirements for cosmetics and food. Here, we determined in vivo/in vitro activities of antioxidant peptide from *P. fucata* (PFAOP) prepared by bio-fermentation method. The antioxidant property test results showed the DPPH, hydroxyl, superoxide radical-scavenging, and cellular antioxidant activity. EC_50_ values of PFAOPs were 0.018 ± 0.005, 0.126 ± 0.008, 0.168 ± 0.005, and 0.105 ± 0.005 mg/ml, respectively, exhibiting higher antioxidant activities than glutathione (*p* < 0.05). Moreover, anti-proliferation and cytotoxicity activity results illustrated PFAOP has a potent anti-proliferative activity against HepG2, Caco-2, and MCF-7 carcinoma cells with no cytotoxicity. Moreover, the protocols we developed in this work demonstrated several excellent advantages in PFAOP preparation compared to enzymatic hydrolysis or chemical synthesis methods and provide a theoretical foundation for higher-value application of marine-derived functional peptides.

## Introduction

Free-radical theory that endogenous oxygen radicals are generated from endogenous metabolic processes in cells and result in a pattern of cumulative damage was proposed by Denham Harman in the mid-1950s [1]. Antioxidant peptides play an important role in our lives because they scavenge free radicals, like superoxide anions or hydroxyls, which damage protein and cause DNA mutations that trigger certain illnesses including coronary heart diseases and diabetes [2]. Typically, macro-molecular proteins do not have antioxidant activity; thus, proteases need to be applied to digest these proteins first. After the protein is enzymatically hydrolyzed, it is decomposed into various small bioactive peptides [3]. Several preparation methods have been developed to obtain antioxidant peptides of high purity and activity including chemical synthesis, enzymatic hydrolysis and bio-fermentation [4, 5]. However, the enzymatic hydrolysis technique has a low extraction rate and is too expensive to be used for antioxidant peptide purification, thereby preventing this method from being widely for industrial production. Meanwhile, the types and amounts of synthesized antioxidant peptides used in food or cosmetics production are very limited because of their potential toxic effects. Thus, most peptides with antioxidant properties are still at their laboratory-scale research stage [3, 5].

Nowadays, marine-derived antioxidant peptides are attracting a lot of attention because of their excellent antioxidant activity, quick physiological absorption, safety, and promising economic value when applied as functional ingredients in cosmetics or health foods [3, 6, 7]. Good example sources of antioxidant peptides include those obtained through the digestion of proteins from marine organisms [8-10]. Meanwhile, several strategies are typically used to enhance antioxidant peptide activity. The first example includes two peptides designed based on glutathione and prepared using L-conformation of each amino acid by solid-phase synthesis. The resulting peptides exhibit high antioxidant activity because of site-directed mutagenesis of the active sites [11]. The second example involves glycosylation used to purify and characterize peptides from *P. fucata* to obtain better antioxidant activity [12]. Other examples are implementation of complex enzymatic hydrolysis to improve antioxidant peptide activity [13] and of pulsed electric field technology to treat four peptides with similar amino acid sequences to enhance their antioxidant activity [14]. Nonetheless, all of these attempts to obtain antioxidant peptides lacked a stable production yield with higher antioxidant activity and purity, attributes necessary for further industrial production.

Recently, an antioxidant peptide from *P. fucata* known to play a protective role during the process of photoaging was prepared and purified using the enzymatic hydrolysis method [15, 16]. However, details on the inhibitory mechanisms against skin photoaging (via molecular docking simulations) of the antioxidant peptide from *P. fucata*, as well as its antioxidant in vivo activity (using cytotoxicity, cellular antioxidant and anti-proliferation activities), were not elucidated, hampering further studies. Therefore, in this paper, we report an antioxidant peptide from *P. fucata* (PFAOP) prepared by bio-fermentation method as a breakthrough for industrialization applications because such peptides can replace the chemically synthesized antioxidants such as butylated hydroxytoluene (BHT), hydroxyanisole (BHA) and others that are used in cosmetics and health foods. Moreover, we also detailed and analyzed the physicochemical characteristics, secondary and tertiary structure and molecular docking simulations of PFAOP to clarify how structure and overall activity are related to each other. Finally, the antioxidant properties of PFAOP, C-PFAOP (antioxidant peptide from *P. fucata* prepared by chemical synthesis method) and E-PFAOP (antioxidant peptide from *P. fucata* prepared by enzymatic hydrolysis method), were analyzed in vitro (using scavenging activity assays of DPPH-, superoxide- and hydroxyl-radicals) and in vivo (using cytotoxicity, cellular antioxidant and anti-proliferation activities) here in our study, the most thorough so far on antioxidant peptides derived from *P. fucata*.

## Materials and Methods

### Chemicals

Glutathione and 2, 2-diphenyl-1-picrylhydrazyl (DPPH) were acquired from Sigma-Aldrich (USA). Expression vector pET-30a (pET) and Rosetta (DE3) strains were preserved in our laboratory. DNA markers 2000, restriction endonucleases (Xho I and Bam HI), DNA Fragment and Plasmid Purification Kits 4.0, DNA gel extraction kits and BCA protein assay were purchased from Takara (Japan). Precast-GL gel Hepes SDS-PAGE, kanamycin, tryptone, isopropyl β-D-1-thiogalactopyranoside (IPTG) and yeast extracts were obtained from Sangon (China). Fetal bovine serum (FBS), protein markers, penicillin-streptomycin solution, Dulbecco’s modified Eagle medium (DMEM) and trypsin-EDTA were purchased from Thermo Fisher Scientific (USA). All commercially purchased chemicals were of analytical grade.

### Bioinformatics Analysis of Antioxidant Peptide Sequence

PFAOP sequence structure was analyzed according to the SWISS-MODEL method by Bertoni, Kiefer, Biasini, Bordoli, & Schwede [17] with some modification. Briefly, the EXPASY database located at https://swissmodel.expasy.org was used to simulate target protein tertiary structures to examine the resulting structural models. Another web site (http://bioinf.cs.ucl.ac.uk/psipred/) was used to predict target protein secondary structure to analyze the relationship between the peptide structure and its antioxidant activity. Theoretical molecular weight, amino acid compositions, isoelectric point, estimated half-life, instability index, hydrophobicity and hydrophilicity scales were obtained for further functional expression and preparation from https://web.expasy.org/protparam/and https://web.expasy.org/protscale/. Moreover, the molecular interaction between PFAOP and porcine elastase (PDB ID: 1ELB) was examined by AutoDock Vina 4.0 according to the reference of Trott & Olson [18] with some modification for virtual screening complex interactions between the receptor 1ELB and antioxidant peptides and to assist with analyzing the potential activity mechanism. A docking grid box (with dimensions of 40×40×40 Å and a certain grid spacing of 0.375 Å) was made to cover the entire binding pocket including the active site. Vina scores (kcal/mol) were calculated with the predicted affinity of peptides difference conformation for binding to the porcine elastase, and the 2D diagram and 3D structure diagram were produced by Discovery Studio 4.5 Visualizer (USA).

### Preparation of Antioxidant Peptide

DNA sequence of PFAOP was codon-optimized for further expression in Escherichia coli system using Primer Premier 5.0 [19] and chemically synthesized by SANGON (China), ligated into the pET30a vector by the Bam HI and Xho I restriction enzymes. Code-optimized sequence of PFAOP was 5’-*ATGCACCATCATCATCATCATTCT TCTGGTCTGGTGCCACGCGGTTCTGGTATGAAAGAAACCGCTGCTGCTAAATTCGAACGCCAGCACATGGACAGCCCAGATCTGGGTACCGACGACGACGACAAGGCCATGGCTGATATCGGATCCCACCATCATCATCATCATAACGGCGGTGCAGGCATTCCGGGTAAACGTGAACGTAATGGTGGTGCCGGTATTCCGGGTAAACGCGAACGTAATGGCGGTGCAGGTATTCCGGGCAAACGCGAACGCAATGGTGGCGCAGGCATTCCTGGCAAACGCGAGCGTAATGGCGGCGCAGGTATTCCTGGTAAACGCGAGCGCAATGGTGGTGCAGGCATCCCGGGCAAACGTGAACGCAATGGCGGCGCCGGCATTCCGGGCAAGCGCGAGAGAAATGGTGGTGCTGGCATTCCGGGAAAACGTGAACGGAATGGCGGCGCGGGTATTCCGGGAAAGCGTGAACGTAACGGCTAACTCGAGTAA*-3’. The positive transformed recombinant engineering strain, the structure of which was confirmed by DNA sequencing, was used for the next induced experimental step and named as DE3-pET-PFAOP.

PFAOP was prepared using a slightly modified method for protein expression and purification reported by Dagar, Adivitiya, & Khasa [20]. For this purpose, DE3-pET-PFAOP was pre-cultured at 37°C in LB medium using a rotary shaker operated at 220 rpm, after which it was grown to a log phase with OD_600_ in the 0.6-0.8 range. It was then induced by 0.5 mM IPTG at 37°C and also cultured for another 12, 6, and 4 h at three different temperatures (20, 30, and 37°C). Cells were centrifuged for 5 min at 5,000 ×*g*, re-suspended in buffer A containing PBS dissolved in glycerine as well as 20% Triton X-100 (v/v), 2 mM DTT at pH = 7.4, and then sonicated for 20 min at 400 W until the solution became colorless. The ultra-sonication regime consisted of 2 s steps alternating with 6 s pauses. The resulting solution was again centrifuged for 20 min at 12,000 ×*g* at 4°C, and the supernatant was discarded. Ni-NTA resin affinity chromatographic column coupled with fast protein liquid chromatographic system (GE Healthcare) was used to purify target protein. All collected fractions were analysed by SDS–PAGE. Protein concentration was measured using BCA protein assay.

### Antioxidant Activity Assay for Antioxidant Peptide

**Scavenging ability towards DPPH radicals.** DPPH radical scavenging activity of PFAOP was determined based on slightly modified method reported in the literature by Xing, Liu, Gao, Zheng, Wang, Zhou, et al. [21]. Experimental group (A_i_) contained antioxidant peptide mixed with 0.2 ml of deionized water and 0.2 ml of 2 × 10^-4^ M DPPH solution in 95% ethanol. The solution was then mixed by vortex mixer and afterwards incubated for 30 min at room temperature in the dark. Absorbance value of the resulting solution was measured at 517 nm using UV-5200 spectrophotometer. Blank group (A_j_) contained just 0.2 ml of 95% ethanol. Control group (A_0_) contained only deionized water. Glutathione, a naturally antioxidant tripeptide with important function in biological systems, was used as a positive control. Scavenging activity towards DPPH radicals was calculated as [1−(A_i_−A_j_)/A_0_] × 100.

**Scavenging activity relative to superoxide radicals.** PFAOP scavenging activity towards superoxide radicals was obtained using a slightly modified method reported in the literature by Wu, Wang, Li, Yang, Wang, & Hu [16]. Briefly, 0.1 ml of peptide was mixed with 2.5 ml of Tris–HCl buffer with pH equal to 8.2 and incubated for 10 min at 25°C, after which 0.1 ml of 3 mM pyrogallic acid was added to the solution. Absorbance of the resulting mixture was recorded at 325 nm every 30 s with the duration of each measurement equal to 5 min. Absorbance of the blank solution, containing deionized water instead of peptide sample, was measured under the same conditions. Glutathione was a positive control test. Scavenging activity towards superoxide radical was calculated as [1−V_i_/V_0_] × 100, where V_0_ and V_i_ are absorbance line slopes of the blank and of the test sample, respectively.

**Scavenging activity towards hydroxyl radical.** PFAOP biological scavenging activity relative to hydroxyl radicals was tested using slightly modified method reported in the literature by Wu, Wang, Li, Yang, Wang, & Hu [16]. Experimental group (A_i_) containing 0.3 ml of PFAOP mixed with 0.1 ml of 3 mM ferrous sulphate, 0.1 ml of 3 mM salicylic acid and 0.1 ml of 3 mM H_2_O_2_ was incubated for 30 min at 37°C and then absorbance was immediately recorded at 510 nm. In control group (A_0_), PFAOP was substituted by deionized water. In blank group (A_j_), salicylic acid was substituted by deionized water. Glutathione was used as a positive control. Peptide scavenging activity relative to hydroxyl radicals was calculated as [1−(A_i_ A_j_)/A_0_] × 100.

### Cellular Antioxidant Activity Assay for Antioxidant Peptide

Cellular antioxidant activity of PFAOP was analyzed using slightly modified method reported by Wolfe & Liu [2]. HepG2 cells used as a cell model were pre-cultured in a cell culture flask at 37°C under atmosphere containing 5% CO_2_. Then the cells were placed into 96-well black plate (2 × 10^5^ cells per well) and incubated again at the same conditions for 24 h. Old culture medium was absorbed and cells were rinsed once with PBS. One hundred microliters of antioxidant peptide dissolved in medium containing 25 mM 2, 7-dichlorodihydrofluorescein diacetate was added and the whole mixture was further incubated for 60 min. Next, the microplate added with 0.6 mM 2,20-azobis(2-amidinopropane) dihydrochloride solution was immediately placed into a Filter Max F5 Multi-Mode Microplate Reader for analysis performed at 535 nm after the solutions in wells were excited at 485 nm every 5 min for 60 min at 37°C. Glutathione was used as a positive control. Cellular antioxidant activity was calculated as 1−∫SA/∫CA, where ∫SA and∫CA are integral areas under the absorbance curves of the test and blank samples, respectively.

### Cytotoxicity Assay and Anti-Proliferation Activity of Antioxidant Peptide

PFAOP cytotoxicity activity and anti-proliferation activity were analyzed by a slightly modified method described in the literature [22-24]. Briefly, the cell culturing method was similar to the method used for the cellular antioxidant activity assay. However, HepG2, Caco-2 and MCF-7 cells pre-cultured as described above were used as model cells. All further incubation and treatment steps were same for all cells. First, cells were seeded at 2 × 10^5^ or 5×10^5^ cells per well in a 96-well microplate for further analysis of anti-proliferation and cytotoxicity activity, respectively. The cells were incubated for 4 h at 37°C under atmosphere containing 5% CO_2_, after which the old culture medium was absorbed and washed with PBS once. Next, 100 μl of peptide solution dissolved in the medium was added to each well and incubated for 24 h. Quantification of viable cells was performed using a Cell Counting Kit-8 (Sangon Biotech Co.). Absorbance was measured at 450 nm.

### Statistical Analysis

PFAOP and glutathione activities related to their radical scavenging, cellular antioxidation, cytotoxicity and anti-proliferation were analyzed using three separate batches. Statistical analysis was conducted using SPSS 13.0 software (SPSS Inc., USA). All values reported in this work represent average values with uncertainties calculated as standard deviations.

## Results

### Bioinformatics Analysis Result

**Fig. 1:**
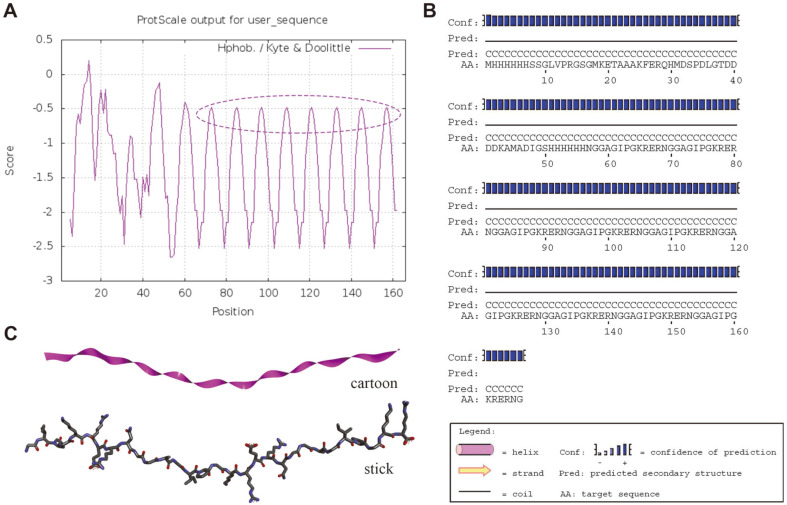
Structural representation depicting PFAOP hydrophobicity (A), secondary structure (B) and 3D spatial conformation (C).

PFAOP chemical formula, instability index (II), theoretical molecular weight and isoelectric point were C_708_H_1155_N_275_O_221_S_4_, 26.52 (stable), 17183.91 Da and 11.04, respectively. The total number of positively (*Arg* + *Lys*) and negatively (*Asp* + *Glu*) charged residues were 32 and 18, respectively. PFAOP half-life in *E. coli* cell was over 10 h. Analysis of the PFAOP amino acid sequence (Fig. 1A) indicated its high hydrophilicity. The primary structure of PFAOP has eight peaks (score < -0.5) at position sections in the 45-100 range. These scales are also often used to judge hydrophobicity or hydrophilicity of the antioxidant peptide. Thus, antioxidant peptides can be easily stored in powder form and easily re-dissolved when needed because of their preserved functional activity provided by amino acid scales. Next, specifics of the secondary PFAOP structure (Fig. 1B) indicate that amino acids should assemble in a random coil structure. Thus, these results suggested that PFAOP had a potential application value in terms of preparation by bio-fermentation using recombinant engineering strains.

To forecast spatial conformation and next-step preparation by bio-fermentation using engineering strains fermentation method, spatial conformation of PFAOP 3D structural representation was constructed by SWISS-MODEL using 2v53.1.B as a template. PFAOP is spatially conformed as a random coil resembled α-helix structure (Fig. 1C). Such a structure could expose active amino acids and their reactive sites toward free radical scavenging, which would result in higher antioxidant activity of the peptide as a whole. Random coil resembling an can also provide data needed to establish the relationship between peptide activity and its spatial conformation.

**Fig. 2:**
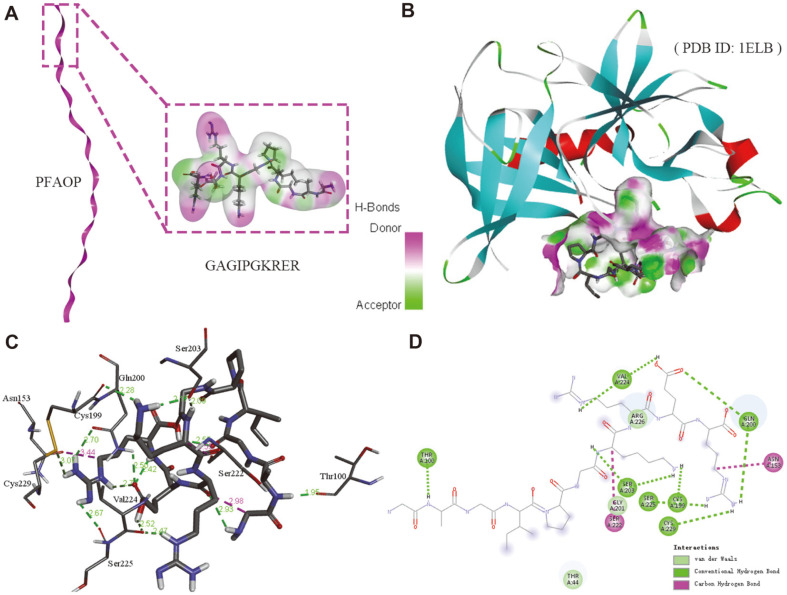
Molecular docking for the interaction of antioxidant peptide PFAOP with porcine elastase (PDB ID: 1ELB). (**A**) Schematic representations of PFAOP and the selective site used for molecule docking, (**B**) Putative binding mode of PFAOP in the binding cavity of 1ELB, (**C**) 3D view of docking pose of PFAOP and 1ELB molecular catalytic site, (**D**) 2Ddiagram of the interaction.

To investigate the interaction between antioxidant peptide PFAOP and porcine elastase 1ELB, molecular docking was carried out. The result (Fig. 2) demonstrated that PFAOP could strongly interact with 1ELB, which displayed the highest affinity to elastase (6.6 kcal/mol). Obviously, twelve hydrogen bonds (including conventional hydrogen bond and carbon hydrogen bond) were observed of PFAOP and the Cys199, Ser203, Val224, Gln200, Cys229, Ser225, Ser203, Thr100, Val224, Asn153, Gln200, and Ser222 residues with a length of 2.3 Å, 2.2 Å, 2.5 Å, 2.7 Å, 3.1 Å, 2.7 Å, 2.1 Å, 1.9 Å, 2.5 Å, 3.4 Å, 2.5 Å, and 3.5 Å, respectively (Figs. 2B-2D). It has been proved that the hydrogen bond interaction force represents a positive effect on the inhibitory activity property of antioxidants. As for GSH (Fig. S1), fewer hydrogen bonds between GSH and 1ELB residues were observed compared to PFAOP, indicating that PFAOP exhibited a higher activity than GSH.

### Validation of PFAOP Preparation

**Fig. 3:**
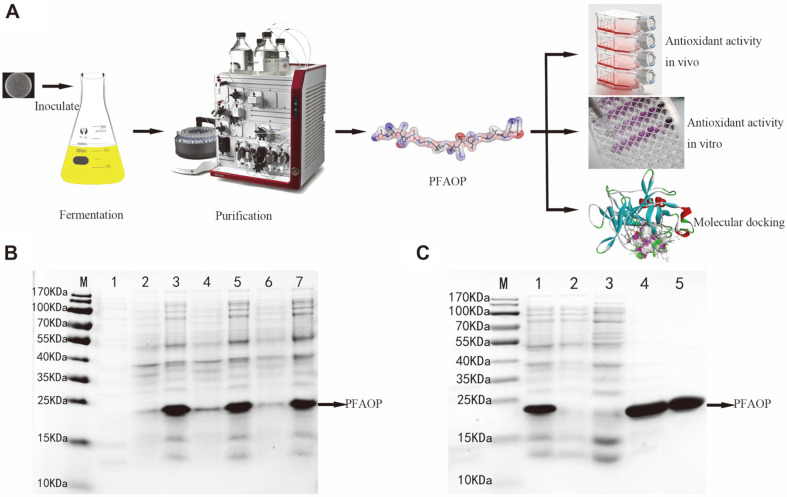
(A) Diagram of preparation using bio-fermentation method for PFAOP; (B) SDS-PAGE analysis of PFAOP at 20, 30, and 37°C. Lane M corresponds to a protein molecular weight marker. Lane 1 corresponds to non-induced lysates. Lanes 2 and 3 correspond to the supernatant induced for 12 h at 20°C, respectively. Lanes 4 and 5 correspond to lysates and to supernatant, respectively, induced for 6 h at 30°C. Lanes 6 and 7 correspond to lysates and to supernatant, respectively, induced for 4 h at 37°C; (C) SDS-PAGE analysis of PFAOP purification process. Lane M corresponds to protein molecular weight marker. Lane 1 corresponds to supernatant of PFAOP induced at 37°C. Lane 2 corresponds to the eluent at the time the sample was loaded. Lane 3 corresponds to rinsing with binding buffer. Lanes 4-5 correspond to eluent containing PFAOP.

Fig. 3A summarizes the preparation using the bio-fermentation method for PFAOP. And this result suggested that this method of preparing PFAOP had some advantages such as cost, degree of automation, preparation speed or scale-up processes compared with the enzymatic hydrolysis or chemical synthesis methods. Preparation of PFAOP was validated in the IPTG absence and presence using 12% SDS–PAGE (Fig. 3B). Only after IPTG induction, a protein band corresponding to molecular weight below 25KD was observed. Comparison of sizes of PFAOP bands with the control (which was non-induced lysate) indicated that molecular weights of target proteins containing designed linker protein sequences corresponded to expected theoretical molecular weights. Thus, successful expression of PFAOP was confirmed. Typically, higher growth temperatures result in increased aggregation and reduced solubility of the heterologous proteins in *E. coli* expression systems. To evaluate PFAOP solubility, we induced expressions at different temperatures using liquid cultures. Fig. 3B showing SDS–PAGE analysis of PFAOP expression indicates protein solubility at all induction temperatures. Only a small difference was observed for inductions of soluble PFAOP performed at 20, 30, and 37°C. Thus, we chose 37°C as the induction temperature to prepare high purity PFAOP for analysis of its in vivo and in vitro antioxidant activities because experiments performed at this temperature require less time and yield protein with higher solubility. After sonication, PFAOP was in the soluble fraction of the supernatant, from which it was extracted by Ni-NTA Resin affinity chromatography column. Results of SDS–PAGE analysis of PFAOP-protein purification showed very good agreement between eluates containing the target protein and its theoretically predicted molecular weight. Only some low-molecular-weight contaminants were observed (Fig. 3C). Purified PFAOP-proteins were obtained after desalination and concentration. Thus, target proteins were successfully prepared by bio-fermentation method using recombinant engineering strains.

### Antioxidant Activity Analysis Results of Antioxidant Peptides

Antioxidant activity of purified PFAOP, C-PFAOP, E-PFAOP and glutathione antioxidants were investigated based on their scavenging activity relative to radicals of DPPH, superoxide and hydroxyl, as well as based on their cytotoxicity and cellular antioxidant activities. The half maximal cytotoxicity concentration (CC_50_) values of PFAOP, C-PFAOP, E-PFAOP and glutathione obtained using cytotoxicity analysis were significantly over 10 mg/ml (Table 1), which is much higher than the corresponding half maximal effect concentration (EC_50_) value, indicating that the inhibitory effects of PFAOP, C-PFAOP, E-PFAOP and glutathione in each case were not attributed to cytotoxic effect. EC_50_ values related to PFAOP antioxidant activity relative to the free radicals of DPPH, hydroxyl, superoxide and its cellular antioxidant activity were 0.018 ± 0.005, 0.126 ± 0.008, 0.168 ± 0.005, and 0.105 ± 0.005 mg/ml, respectively (Table 1). Obviously, the analogous EC_50_ values of antioxidant activity of glutathione (a common cosmetics ingredient also used in this study as a positive control) were lower (*p* < 0.05): 0.030 ± 0.006, 0.318 ± 0.012, 0.392 ± 0.015, and 0.128 ± 0.009 mg/ml, respectively, and a similar trend was observed for C-PFAOP and E-PFAOP. Thus, PFAOP had the highest antioxidant activity during radical scavenging and cellular antioxidant activity experiment, followed by glutathione, E-PFAOP and C-PFAOP. In particular, the hydroxyl radical scavenging activity of E-PFAOP (the EC_50_ value was 0.322 ± 0.022 mg/ml) was not significantly different from that of glutathione (the EC_50_ value was 0.318 ± 0.008 mg/ml) (*p* > 0.05).

### Anti-Proliferation and Cytotoxicity Analysis of Antioxidant Peptides

Anti-proliferation and cytotoxicity activities of PFAOP, C-PFAOP, E-PFAOP and glutathione antioxidants against HepG2, Caco-2 and MCF-7 cells expressed as EC_50_ and CC_50_ are shown in Table 2. PFAOP EC_50_ values with regard to HepG2, Caco-2 and MCF-7 cells were 0.069 ± 0.008, 0.145 ± 0.012, and 0.182 ± 0.009 mg/ml, respectively. Particularly, PFAOP had the highest inhibitory effect against HepG2 cell proliferation, followed by Caco-2 and MCF-7 cells. A similar trend was observed for glutathione, C-PFAOP and E-PFAOP, but the corresponding values indicated higher anti-proliferation activity of PFAOP than the other three (*p* < 0.05). Cytotoxicity activity analysis of PFAOP, C-PFAOP, E-PFAOP and glutathione showed that corresponding CC_50_ values were significantly higher than their EC_50_ values. Thus, PFAOP had better anti-proliferative ability against HepG2, Caco-2 and MCF-7 cells with no cytotoxicity.

### PFAOP Stability

**Fig. 4:**
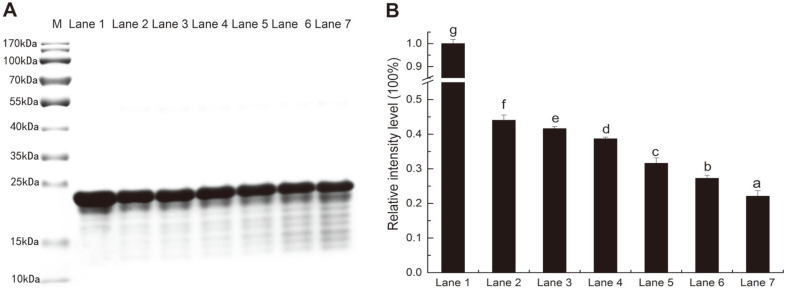
SDS-PAGE analysis results (**A**) and relative intensity (**B**) of PFAOP at 0, 6, 30, 60, 120, 240, and 360 min (Lanes 1 through 7, respectively). Lane M corresponds to the marker. Letters a through g in (**B**) indicate significant differences (*p* < 0.05).

Antioxidant activity stability and content changes of purified PFAOP during its antioxidant reaction process as well as clarification of the relationship between spatial conformation structure and antioxidant activity were determined based on analysis of samples taken at different periods during total antioxidant capacity experiment performed using 12% SDS-PAGE (Fig. 4A). PFAOP content decreased as reaction time increased, and relative intensity of PFAOP was continuously decreasing (*p* < 0.05) during total antioxidant capacity test (Fig. 4B). In addition, PFAOP free radical scavenging activity time was over 360 min, which indicates that more free radicals were neutralized with reaction time increased. This can be explained by the excellent stability of the PFAOP spatial conformation structure and exposure of more binding sites for effective neutralization of free radicals.

## Discussion

Differences between the enzymatic hydrolysis, bio-fermentation and chemical synthesis methods occur mostly whether spatial conformation exists of antioxidant peptide from *P. fucata*: L-conformation type amino acids are commonly selected to synthesize for the chemical synthetic method, while L-conformations and R-conformations (like the formation of a random coil or other spatial conformation structures) were both observed during antioxidant peptides preparation when using enzymatic hydrolysis and bio-fermentation methods [5, 25]. Certainly, antioxidant peptides could also be chemically synthesized using amino acids with L- and R-conformations. However, if every amino acid site in a peptide chain could freely be in either L- or R-conformation, then millions of antioxidant peptide chains should be synthesized to obtain the appropriate spatial conformation [26]. Likewise, the enzymatic hydrolysis method, which is another generally used preparation method, requires substantial effort and materials as well as time and temperature optimization for effective enzyme hydrolysis to meet the needs of industrial production applications. Additionally, compared with common separation and puriﬁcation methods (*e.g.*, chemical synthesis and enzymatic hydrolysis methods), the bio-fermentation method is versatile and flexible enough to be implemented for large-scale production purposes as well as for structural modiﬁcation of antioxidant peptides [5]. Furthermore, bio-fermentation protocol also enables rapid expression and high yield, is inexpensive and is very simple to scale-up to prepare and purify antioxidant peptides [20, 27, 28].

Previous literature reports some antioxidant peptides from *P. fucata* organisms that were isolated, puriﬁed, and characterized in vitro using enzymatic hydrolysis and chemical synthesis methods [15, 16, 29]. However, these antioxidant peptides are still at the laboratory-scale research stage due to their low yield or low antioxidant activity, which does not meet the requirements for industrial production of cosmetics and health food. Before this work, antioxidant peptides such as glutathione or L-carnosine with less than three amino acids were commonly used additives in the cosmetics industry instead of chemical antioxidants such as BHT and BHA, but only a few studies reported on antioxidant peptides with more than 10 amino acids [4, 30, 31]. So, preparation of antioxidant peptides from *P. fucata* using the bio-fermentation method could establish a new innovative way to provide a breakthrough and reach the next level of industrialization application as with glutathione.

In this work, antioxidant peptides from *P. fucata* of PFAOP were prepared and characterized using bio-fermentation method. As shown in Fig. 3, PFAOP was successfully expressed and purified by IPTG induction at ﬁnal concentration equal to 0.5 mM. Obviously, purified PFAOP was stable and soluble in this research, and this result was in agreement with other reports on bioactivity peptides prepared using bio-fermentation technology of recombinant engineered strains [27]. These results provide data for future research of potential PFAOP applications because PFAOP and glutathione are both water-soluble. Meanwhile, analysis of physicochemical properties, secondary and tertiary structure of PFAOP allowed us to establish connection between its structure and antioxidant characteristics. We discovered a secondary coil structure (Fig. 1B) that plays an important role in PFAOP functional activity compared with its peptide chain length, degree of side chain amino acid glycosylation and sizes as well as with its molecular weight. Moreover, the stick model of PFAOP was obtained by SWISS-MODEL analysis using 2v53.1.B as a template (Fig. 1C), indicating that PFAOP had spatial conformation shaped as a randomly-formed coil, which is capable of exposing more active-sites of amino acid residues to scavenge free radicals. We therefore believe that the more the coil formed, the more amino acid residues became affected by the driving force responsible for higher activity (Table 1); however, this will require additional research so that the coil structure can be characterized using nuclear magnetic resonance, cryo-electron microscopy or crystallography method [5, 25, 32]. Besides, molecular docking is an effective method for evaluating the interaction between ligand PFAOP and receptor1ELB, and the higher elastase inhibitory activity of PFAOP might result from its stronger intermolecular interaction with elastase, which was simulated and calculated by AutoDock Vina 4.0 software compared with that of GSH. To sum up, the antioxidant activity of PFAOP was not only related to its amino acid sequence, peptide chain length, amino acid composition, molecular weight and so on, but also to secondary structure and spatial conformation structure. This study can provide data to understand how peptide activity correlates with its coil structure for clarifying the relationship between activity and structure, as well as to establish a novel path for the next level of industrial applications.

Moreover, PFAOP activities related to free radical scavenging, cytotoxicity, cellular antioxidation and anti-proliferation were examined to evaluate its overall antioxidant properties, as well as provide test data to clarify the relationship between structure and activity. A comparison of the antioxidant activities in vivo and in vitro of PFAOP, C-PFAOP, E-PFAOP and glutathione (Table 1) illustrated that glutathione, C-PFAOP, E-PFAOP and PFAOP had bioactivity properties. PFAOP exhibited higher scavenging activity towards free radicals of DPPH, OH and superoxide, as well as higher cellular antioxidant activity than that of glutathione (*p* < 0.05). A similar trend is seen in C-PFAOP and E-PFAOP. Among them, the DPPH scavenging free radicals increased by about 1.7 times compared with glutathione which is a common natural antioxidant tripeptide used in cosmetics applications. Similarly, EC_50_ value of PFAOP was 0.018 mg/ml, thus, the DPPH scavenging free radicals increased by about 7.5 times compared with E-PFAOP, which was prepared using the enzymatic hydrolysis method, as well as 20.2 times compared to C-PFAOP prepared by chemical synthesis method. Moreover, EC_50_ values related to scavenging activity of antioxidant peptides from *Oreochromis niloticus* and from salmon towards DPPH radicals were 0.6 mg/ml [33] and 1.63 mg/ml [34], respectively (*p* < 0.05). Meanwhile, PFAOP also had superoxide scavenging activity superior to other marine-derived antioxidant peptides: corresponding EC_50_ values for antioxidant peptides from blue mussel [35] and Blueﬁn leatherjacket [36] were 0.228 mg/mL and 2.881 mg/mL, respectively (*p* < 0.05). In addition, the superoxide scavenging free radicals increased by about 5.6 times compared with E-PFAOP, as well as 6.3 times compared to C-PFAOP. Similarly, the superoxide scavenging free radicals increased by about 2.3 times compared with glutathione. Moreover, the hydroxyl scavenging free radicals increased by about 2.5 times compared with glutathione, and PFAOP also demonstrated the highest value for scavenging of hydroxyl free radical reported in the recent literature, such as OH scavenging activity of antioxidant peptides from loach protein [37] and *Oreochromis niloticus* [33], which demonstrated EC_50_ equal to 2.64 mg/ml and 0.263 mg/ml, respectively (*p* < 0.05). Particularly, the hydroxyl scavenging free radicals increased by about 2.5 times compared with E-PFAOP, as well as 7.5 times compared to C-PFAOP. All these results confirmed PFAOP bio-computing analysis on coil structure (Fig. 1B) and molecular docking (Fig. 2C) which could provide more targeted binding sites for radical scavenging activity in vitro experiments. Thus, all tests on radical scavenging activity revealed the high potency of PFAOP, which makes it a very valuable, novel functional oligopeptide suitable for industrial applications in cosmetics and health foods.

Additionally, PFAOP exhibited higher in vitro radical scavenging activity, and the same is true for cell cellular antioxidant activity and anti-proliferation activity in vivo experiments. Judging by the EC_50_ values of cellular antioxidant and cytotoxicity activities (Table 1), PFAOP showed better cellular antioxidant activity than glutathione, C-PFAOP and E-PFAOP with no cytotoxicity with EC_50_ value equal to 0.105 ± 0.005 mg/ml (*p* < 0.05). Typically, EC_50_ is the concentration calculated from the median effect curve, and the lower EC_50_ values, the higher the cellular antioxidant activity is [2, 22]. So, PFAOP had better cellular antioxidant activity than reported antioxidant peptides from highland barley with EC_50_ equal to 3.79 mg/ml (*p* < 0.05) [38]. Moreover, PFAOP cytotoxicity and anti-proliferation activities, tested against HepG2, Caco-2 and MCF-7 carcinoma cells, showed highest inhibition against HepG2 cell proliferation, followed by Caco-2 and MCF-7 carcinoma cells (Table 2). This is consistent with the literature reporting that if compounds can remove reactive oxygen species in carcinoma cells, they may inhibit the cell growth [20, 22, 38]. PFAOP was also more effective against proliferation of all cells tested in this work than glutathione (*p* < 0.05), and this result confirmed PFAOP bio-computing analysis on molecular docking (Fig. 2D) which formed more hydrogen bonds, van der Waals forces and carbon hydrogen bonds than that of GSH. All these results confirmed PFAOP bio-computing analysis on coil structure (Fig. 1B) and molecular docking (Fig. 2C) which could provide more targeted binding sites for radical scavenging activity in vitro experiments. Thus, PFAOP could inhibit proliferation of HepG2, Caco-2 and MCF-7 cells more effectively than glutathione, C-PFAOP and E-PFAOP even at low PFAOP concentrations very likely because of its structure-activity relationship such as spatial conformation structure, which needs to be studied in detail in the future by nuclear magnetic resonance, cryo-electron microscopy or crystallography technology. Furthermore, PFAOP stability, including in vitro and in vivo, possesses a very important advantage over other functional marine-derived oligopeptides (*e.g.*, glutathione and L-carnosine) when considered for practical applications in cosmetics [5, 32]. Reliable stability and long-lasting antioxidant activity of PFAOP (Fig. 4) make it appropriate for a wider range of cosmetics industrial applications and mass production, and this result was confirmed in PFAOP bio-computing analysis on instability index (II) 26.52, which demonstrates the stable and spatial conformation structure that could provide more targeted binding sites for scavenging free radicals.

In conclusion, we prepared antioxidant peptides of PFAOP using bio-fermentation method in this work. Unlike traditionally separation and puriﬁcation techniques (*e.g.*, enzymatic hydrolysis and chemical synthesis), the method used in this work was simpler, required less time and produced higher yield. Analysis of antioxidant activity in vivo and in vitro of PFAOP, which was relative to cellular antioxidant activity and free radicals of superoxide, DPPH, and OH showed significantly higher in vitro and in vivo activities than for glutathione, C-PFAOP and E-PFAOP (*p* < 0.05). Besides, PFAOP exhibited an efficient anti-proliferative activity when it was tested against HepG2, Caco-2 and MCF-7 carcinoma cells. Molecular docking simulations of PFAOP were studied for better understanding the relationship between structure and activity. In summary, the proposed bio-fermentation method which obtained antioxidant peptides had a stable production yield with higher antioxidant activity and purity, and will increase the availability of PFAOP for research and its potential applications in cosmetics and health food industries. It can also serve as a basis and data for further property analysis and downstream applications of other functional peptides derived from marine resources.

## Supplemental Material

Supplementary data for this paper are available on-line only at http://jmb.or.kr.

## Figures and Tables

**Table 1:** EC_50_ values (in mg/ml) for DPPH, hydroxyl and superoxide radical scavenging and cellular antioxidant activities as well as CC_50_ value (in mg/ml) for cytotoxicity of PFAOP, C-PFAOP, E-PFAOP and glutathione antioxidants.

Antioxidant type	Scavenging activity against free radicals of: (EC_50_, mg/ml)			Cellular antioxidant activity (EC_50_, mg/ml)	Cytotoxicity (CC_50_,mg/ml)

	DPPH	Hydroxyl	Superoxide		
PFAOP	0.018±0.005a	0.126±0.008a	0.168±0.005a	0.105±0.005a	>10
C-PFAOP	0.365±0.016d	0.955±0.062c	1.072±0.053d	1.369±0.075d	>10
E-PFAOP	0.136±0.007c	0.322±0.022b	0.946±0.036c	0.985±0.036c	>10
Glutathione	0.030±0.006b	0.318±0.008b	0.392±0.015b	0.128±0.009b	>10

Values are shown as average of three measurements with uncertainties calculated as standard deviation of the mean: average ± SD (*n* = 3). EC_50_ and CC_50_ are half maximal effect and cytotoxicity concentrations, respectively. Letters a, b, c, and d indicate significant difference (*p* < 0.05).

**Table 2:** Anti-proliferative (EC_50_) and cytotoxicity (CC_50_) activities (both in mg/ml) of PFAOP, C-PFAOP, EPFAOP and glutathione antioxidants against HepG_2_, Caco-2, and MCF-7 cells.

Variety	HepG_2_		Caco-2		MCF-7	

	EC_50_(mg/ml)	CC_50_(mg/ml)	EC_50_(mg/ml)	CC_50_(mg/ml)	EC_50_(mg/ml)	CC_50_(mg/ml)
PFAOP	0.069±0.008a	>10	0.145±0.012a	>10	0.182±0.009a	>10
C-PFAOP	1.365±0.037d	>10	1.562±0.038d	>10	1.706±0.049d	>10
E-PFAOP	1.056±0.035c	>10	1.264±0.051c	>10	1.525±0.065c	>10
Glutathione	0.125±0.011b	>10	0.343±0.007b	>10	0.387±0.015b	>10

Values are shown as average of three measurements with uncertainties calculated as standard deviation of the mean: average ± SD (*n* = 3). EC_50_ and CC_50_ are half maximal effect and cytotoxicity concentrations, respectively. Letters a, b, c, and d indicate significant difference (*p* < 0.05).
