# Supplementary material for: In Vivo/In Vitro Properties of Novel Antioxidant Peptide from Pinctada fucata
Source: J Microbiol Biotechnol. 2020 Jul 30;31(1):33–42. doi: 10.4014/jmb.2006.06002 (PMC9705889; doi:10.4014/jmb.2006.06002)
Supplement: Supplementary file 1 [file jmb-31-1-33-supple.pdf]

J. Microbiol. Biotechnol.
https://doi.org/10.4014/jmb.2006.06002

Appendix A

Supplementary data:

Fig. S1.

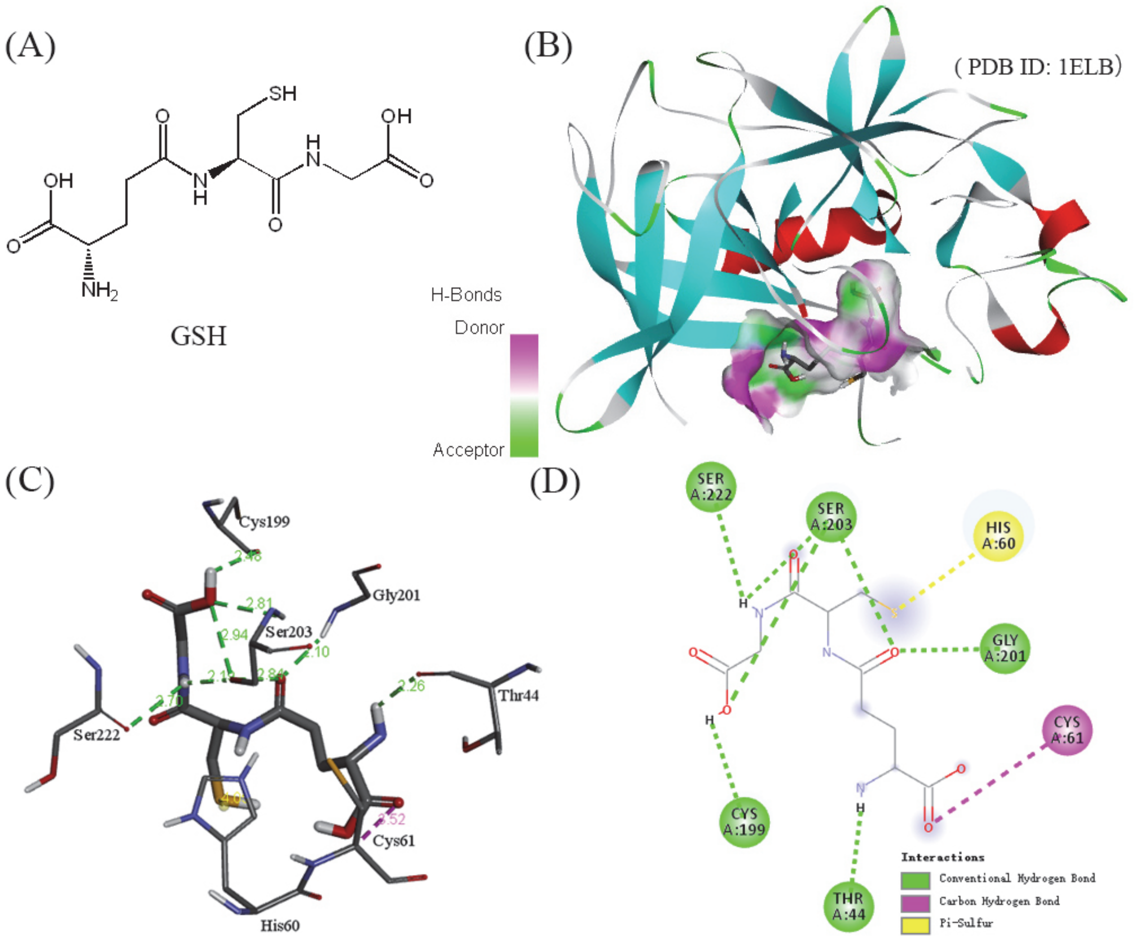

Fig. S1. Molecular docking for the interaction of GSH with porcine elastase (PDB ID: 1ELB). (A) Schematic representations of GSH, (B) Putative binding mode of GSH in the binding cavity of 1ELB, (C) 3D view of docking pose of GSH and 1ELB molecular catalytic site, (D) 2D-diagram of the interaction.
